# The Evolution of Collective Restraint: Policing and Obedience among Non-conjugative Plasmids

**DOI:** 10.1371/journal.pcbi.1003036

**Published:** 2013-04-18

**Authors:** Kyriakos Kentzoglanakis, Diana García López, Sam P. Brown, Richard A. Goldstein

**Affiliations:** 1 Division of Mathematical Biology, MRC National Institute of Medical Research, London, United Kingdom; 2 School of Physics and Astronomy, University of Manchester, Manchester, United Kingdom; 3 Centre for Immunity, Infection and Evolution, School of Biological Sciences, University of Edinburgh, Edinburgh, United Kingdom; University of New South Wales, Australia

## Abstract

The repression of competition by mechanisms of policing is now recognized as a major force in the maintenance of cooperation. General models on the evolution of policing have focused on the interplay between individual competitiveness and mutual policing, demonstrating a positive relationship between within-group diversity and levels of policing. We expand this perspective by investigating what is possibly the simplest example of reproductive policing: copy number control (CNC) among non-conjugative plasmids, a class of extra-chromosomal vertically transmitted molecular symbionts of bacteria. Through the formulation and analysis of a multi-scale dynamical model, we show that the establishment of stable reproductive restraint among plasmids requires the co-evolution of two fundamental plasmid traits: policing, through the production of plasmid-coded trans-acting replication inhibitors, and obedience, expressed as the binding affinity of plasmid-specific targets to those inhibitors. We explain the intrinsic replication instabilities that arise in the absence of policing and we show how these instabilities are resolved by the evolution of copy number control. Increasing levels of policing and obedience lead to improvements in group performance due to tighter control of local population size (plasmid copy number), delivering benefits both to plasmids, by reducing the risk of segregational loss and to the plasmid-host partnership, by increasing the rate of cell reproduction, and therefore plasmid vertical transmission.

## Author Summary

Mutual policing constitutes an important mechanism for the emergence and maintenance of cooperation through the repression of intra-group competition among a population of self-interested individuals. Existing models of mutual policing have been highly abstract and distant from the properties of real biological systems. In this paper, we construct a bottom-up, multi-scale computational model reflecting the biology of, perhaps, the simplest example of such a mechanism: replication control in non-conjugative plasmids, a class of vertically transmitted, molecular symbionts of bacteria. We simulate the emergence of plasmid copy number control through the co-evolution of two interacting plasmid traits: policing, realized as the production of trans-acting replication inhibitors, and obedience, expressed as plasmid-inhibitor binding affinities. We demonstrate and explain the intrinsic replication instabilities that arise in the absence of policing and we show how increasing levels of policing and obedience resolve these instabilities and improve both plasmid stability and host performance.

## Introduction

The evolution of cooperation is a fundamental problem in biology: why help another individual to reproduce, if this comes at a cost to one's own reproductive success? This dilemma is reflected in the trade-off between an individual's immediate reproductive gains and its longer-term prospects of success as part of a collective, whose stability and overall performance is undermined by internal competitiveness. The eroding consequences of competition are exemplified by the “tragedy of the commons” [1], in which a common resource is over-exploited and eventually destroyed by a group of self-interested individuals who act in order to maximize their immediate personal yield from that resource. The conflict between individual and group interests, however, does not prevent the emergence of cooperation: from genes on genomes and chromosomes in cells, to multicellularity, eusociality and beyond, harmonious cooperative behavior is both widespread and persistent across all levels of biological complexity. The mechanisms underlying the moderation of individual competitiveness (self-restraint), as a means of promoting cooperation, rely on the social interactions between individuals within a group. Sufficiently high genetic relatedness among interacting individuals can promote intra-specific cooperation and the evolution of self-restraint through the carriage and transmission of shared “cooperative” genes [2]. However, kin selection can not account for the maintenance of costly cooperation when individuals are distantly related or even not related at all, in which case various forms of reciprocity can support cooperative behavior by assuring direct fitness returns to focal actors (for reviews, see [3]–[5]). At low relatedness, the repression of internal competition for the benefit of the collective can also be achieved through individual investment in appropriate enforcement mechanisms such as mutual policing, resulting in a level-playing field within the group that motivates individuals to contribute towards the enhancement of the group's efficiency and productivity in order to increase their own reproductive success [6]–[9].

A particularly elegant example of a policing mechanism for the repression of competition among individuals within a group is the replication control system of bacterial plasmids. Plasmids are extra-chromosomal DNA elements, organized as, typically circular, collections of discrete genetic modules [10], [11], including those encoding functions necessary for their survival and propagation such as self-replication and its control, active partitioning during cell division, and conjugative transfer. Plasmids replicate autonomously by making use of the replication machinery of their host; they also encode a policing mechanism for controlling their replication [12], [13]. The role of this mechanism is to ensure that each plasmid copy replicates once per cell cycle on average, so as to maintain a stable characteristic copy number under constant conditions. In plasmid R1 for example, copy number control (CNC) is achieved through the constitutive synthesis of trans-acting replication inhibitors, in the form of the widely used plasmid-coded antisense RNAs [14], that decay rapidly so that their concentration is proportional to the plasmid's copy number [15], [16]. Inhibitors act by binding to and deactivating a plasmid-specific target that is rate-limiting for the initiation of plasmid replication. The presence of inhibitors induces the establishment of a negative feedback loop between the plasmid copy number and individual plasmid replication rates: higher copy numbers result in a higher concentration of trans-acting inhibitors in the cell, thereby effecting a reduction in the frequency of plasmid replication and vice versa.

The plasmid CNC mechanism encapsulates the two fundamental traits of standard generic policing models [6]–[9], [17], namely individual competitiveness (selfishness) captured by the production of rate-limiting constitutive factors (such as Rep proteins [18], [19] or RNA primers [20]) that are responsible for the initiation of plasmid replication, and mutual policing captured by the synthesis of trans-acting replication inhibitors that act upon the target initiation factor, thus mediating the repression of competition in the plasmid replication pool. In addition to these two fundamental traits, the mechanistic structure of the CNC system motivates the consideration of a third one: the obedience of individuals to the policing resources that they themselves produce, as expressed by the binding affinity of designated inhibitor targets on individual plasmids to the inhibitor molecules. The distinction between contributing towards the production of policing resources (by producing generic trans-acting inhibitors) and being sufficiently obedient to policing (by appropriately responding to these inhibitors) creates the potential for subversive strategies: an individual plasmid can gain a competitive advantage in the intra-cellular replication pool by contributing towards the collective production of policing resources, provided that its own sensitivity to these resources is lower than the sensitivity of its coresident plasmids. Nevertheless, the good of the collective (host cell) can prevail over the short-sighted self-interest of individual plasmids, provided that, first, there is limited migration of individuals between collectives (i.e. slow rate of horizontal transmission); second, each new collective is founded by a limited number of collectives (e.g. each daughter cell results from binary fission of a single parent cell); and, third, the number of collectives exceeds the number of individuals per collective (i.e. host population size far exceeds the per-host plasmid copy number) [21].

The CNC mechanism for the collective restraint of plasmid selfishness via mutual policing operates in a clear inter-specific context: plasmids often code for accessory adaptive traits that can provide their hosts with a variety of competitive selective advantages under particular environmental conditions [22]. Examples include resistance to antibiotics and heavy metals, the ability to exploit new niches and to metabolize unusual environmental elements, the capacity to synthesize toxins and virulence factors etc. However, the carriage of locally beneficial allele(s) is not always sufficient to guarantee a mutualistic outcome in the host-plasmid relationship, since the host also bears the metabolic cost of plasmid maintenance, due to the plasmids' usage of the cellular machinery for the purpose of gene expression, replication etc. The cost of plasmid carriage is positively correlated with the copy number [23]–[25] and if the number of plasmid copies within the focal cell is such that the metabolic costs accociated with plasmid maintenance exceed the benefits of the focal trait then the mutualistic host-plasmid relationship degrades to parasitism.

The policing mechanism for the control of plasmid replication is subject to a dynamic evolutionary conflict between two levels of selection [26], [27]: at the intra-cellular level, plasmid mutations that induce an increased rate of initiation of plasmid replication (selfishness) or a reduced binding affinity to the trans-acting inhibitor (obedience) would result in plasmids that proliferate faster in comparison to more frugal plasmids with a higher degree of adherence to the CNC mechanism. Hence, intra-cellular selection will favor plasmid selfishness and oppose obedience to collective policing. The consequent escalating drive towards the ratcheting of plasmid replication rates is an example of a tragedy of the commons, as hosts inhabited by selfish and disobedient plasmids become increasingly unable to bear the metabolic burdens associated with the elevated copy numbers. However, such hosts find themselves in a disadvantageous position compared to fellow hosts inhabited by more obedient plasmids, where stricter replication control results in more moderate metabolic costs. This way, selective pressures towards plasmid recklessness at the intra-cellular level are counter-balanced by inter-cellular selection that penalizes hosts with disobedient plasmids and, therefore, disobedient plasmids themselves. The pressure for effective CNC is particularly strong for non-conjugative plasmids, i.e. purely vertically-transmitted symbionts that lack the capacity for horizontal (infectious) transmission to neighboring hosts. By forgoing horizontal transfer, vertically transmitted symbionts hook their reproductive fate to that of their hosts, thus forging an alliance of fitness interests that can support an elaborate mutualistic host-symbiont relationship [28], [29].

In this paper, we explore a range of social dilemmas facing non-conjugative plasmids carrying beneficial alleles. These dilemmas are present in the mechanistic interactions between selfishness, policing and obedience that determine the efficiency of the CNC system. More specifically, we use a mathematical model of the symbiotic relationship between hosts and plasmids in order to investigate how the conflict between intra-cellular selection (favoring plasmid recklessness with respect to replication) and inter-cellular selection (favoring an optimal metabolic balancing of costs and benefits in hosts) orchestrates the evolution of the plasmid-coded CNC mechanism.

## Models

### Cell Growth

Our model for cellular growth and division (or death) is based on the premise that cell metabolism produces biomass and when this increasing mass reaches a certain threshold the cell divides. Specifically, let 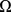 be the cell's biomass, with an initial value 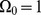, which is updated at every time interval 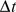 according to 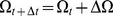. When the biomass doubles, i.e. when 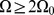, the cell divides. Inefficiencies in cell metabolism can lead to negative growth (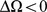) and, eventually, to death when the biomass 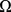 of a shrinking cell falls below zero.

**Figure 1:**
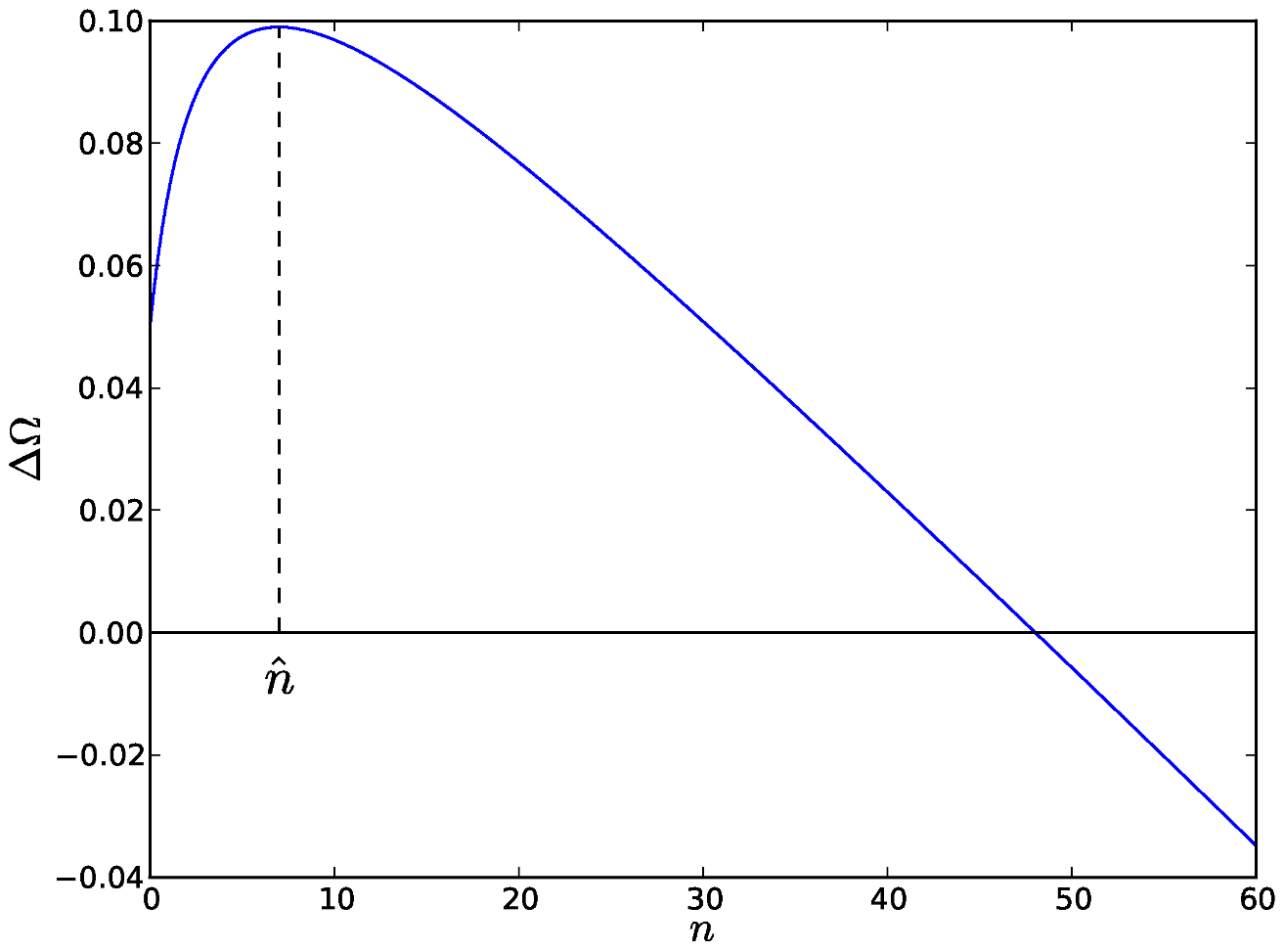
Effect of plasmid copy number on cellular growth. Cellular growth rate 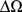 as a function of the plasmid copy number 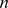 (following Equation 1), with 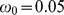, 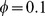, 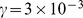 and 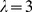. The copy number 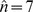, which is optimal for cell growth, is denoted by the vertical dashed line.

Changes in biomass 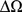 are determined by the chromosomal and plasmid contributions to cellular metabolism, as well as the metabolic costs of plasmid maintenance paid by the host. We consider a plasmid type, which has a positive contribution to host growth that saturates with copy number 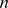 and converges towards a value 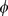 (a saturating gene dosage effect). In essence, 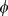 measures the strength of the selective pressure for the plasmid trait in the homogeneous environment of the cellular population. Let 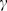 be the general cost of maintaining a single plasmid copy, including the costs of gene expression, plasmid replication etc. We define the rate of cell growth, expressed as the change 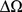 in biomass per unit time step, according to:
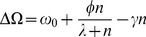
(1)where 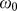 is the metabolic contribution of the cell's chromosome or the cell's basal growth rate and 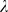 characterizes the steepness of the curve that describes the saturating beneficial contribution of the plasmid to host growth as a function of the copy number 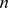. The form of 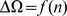 implies that there exists a copy number value 
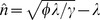
 for which the positive offset between the plasmid's contribution to cell growth 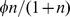 and the plasmid's burden to cell metabolism 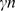 is maximum, resulting in an optimal cellular growth rate (see Figure 1). As such, cells that sufficiently deviate from the average copy number 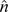 that is optimal for their growth, due to the increasing recklessness of their resident plasmids, will be penalized by slower growth rates, while cells that do not will be rewarded with faster growth rates. In effect, Equation 1 establishes the dependence of the cellular growth rate on the plasmid copy number, which varies over the cell cycle as plasmids replicate autonomously within the host, a process to which we now turn our attention.

### Plasmid Replication

A wide range of mathematical models of varying specificity and complexity have been proposed for describing the autonomous replication of plasmids within a host, mostly based on the replication systems of plasmids R1 and ColE1 [15], [27], [30]–[34]. We adopt a generic approach, according to which an unstable plasmid-coded trans-acting replication inhibitor (e.g. in the form of antisense RNA) binds to and deactivates a plasmid-specific target that participates in the initiation process, thereby down-regulating the individual plasmid replication rate [35]. Each plasmid 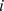 is, first, characterized by a basal replication rate 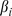, which indicates the plasmid's replication rate in the absence of any copy number control system. Each plasmid 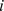 also has a binding affinity 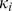 to a trans-acting, generic replication inhibitor, which is synthesized by all plasmids at a rate 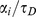 per plasmid, where 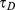 is the (short) averarge lifetime of the inhibitor, resulting in a total inhibitor concentration of 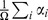 in the host, as the inhibitor is diluted with increasing biomass 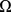. The weighted response of plasmid 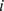 to the collectively produced inhibitor modulates its basal replication rate and yields the actual plasmid replication rate 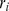 according to:
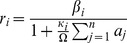
(2)where 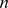 is the number of plasmids in the host. As the binding affinities are constrained by the limits of physico-chemical interactions, we set 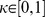. Similarly, we assume that the basal replication rate 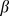 and the rate 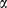 of inhibitor production are limited by physiological and biochemical constraints, therefore we also set 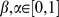. Our model also assumes that there is no influence of the host growth rate on the capacity of plasmids to replicate. If that were the case, it would effectively function as a secondary mechanism for CNC.

Parameters 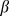 (baseline reproduction rate) and 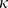 (obedience) of a given plasmid act in cis, since their values influence only the replication rate of the plasmid itself, while 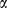 (policing) acts in trans, since its value influences the replication rate of every plasmid within the host through the aggregate term 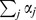. Plasmid mutations inducing a higher basal replication rate 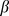 or a lower responsiveness 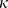 to the collectively produced inhibitor are favored by intra-cellular selection due to the consequent increase of the mutant's replication probability. This drive towards ever-increasing plasmid recklessness is counterbalanced by selection acting at the level of host cells and creates the context within which the evolution of CNC can be investigated.

## Results

### The Existence of Characteristic Copy Numbers

**Figure 2:**
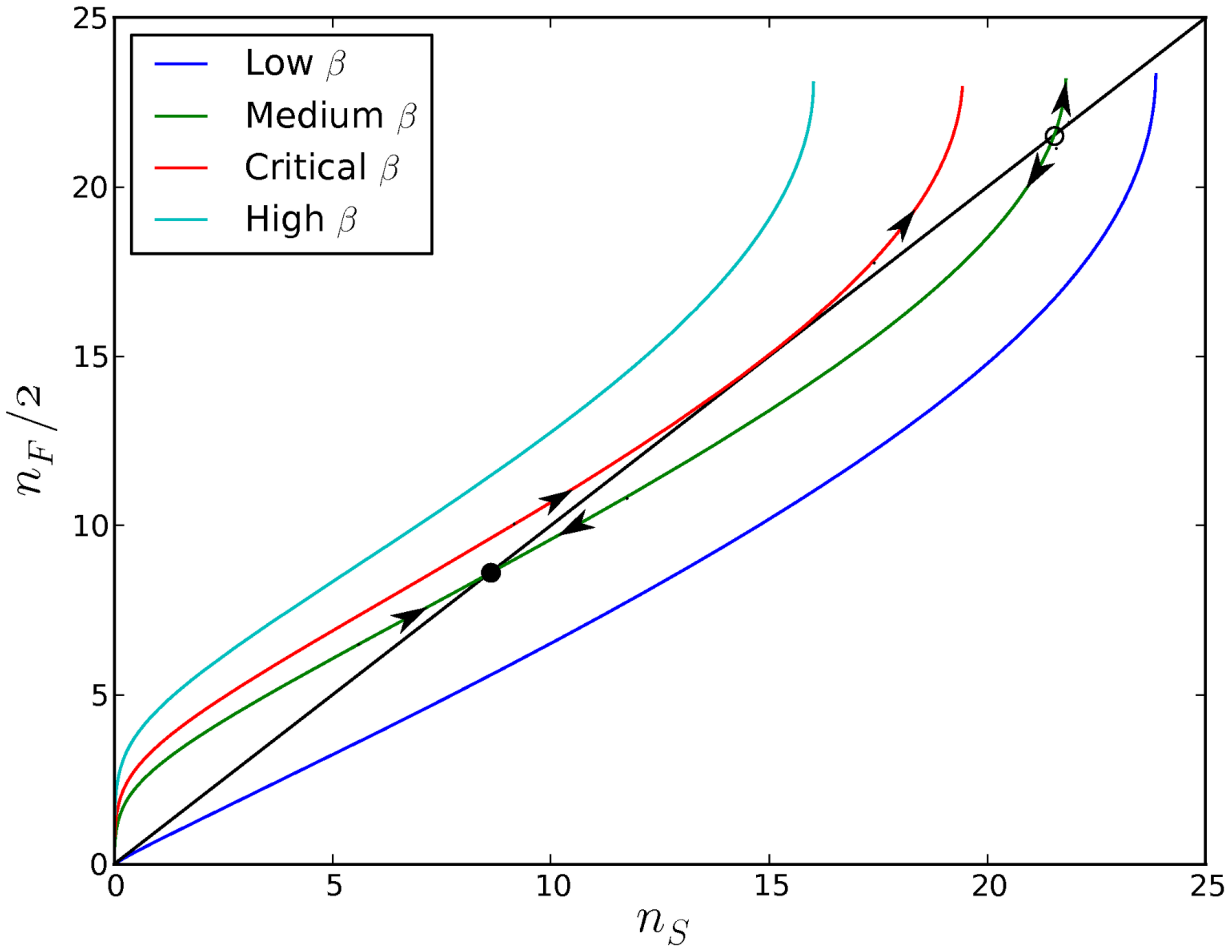
Inter-generational copy number stability. Each curve represents the deterministic relationship between the number of plasmids 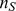 at the beginning of the parent cell cycle and the number of plasmids 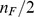 at the beginning of the daughter cell cycle (assuming equipartitioning during cell division), for a range of initial copy numbers 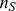 and a fixed set of cell and plasmid parameters. The diagonal represents the consistency of copy numbers between parent and daughter cells; points below the diagonal indicate plasmid under-replication, while points above the diagonal indicate plasmid over-replication. The curve's shape and its point(s) of intersection with the diagonal depend on the plasmid parameter values; curves that do not intersect with the diagonal represent the case of consistent under- or over- replication of plasmids for any initial copy number (blue low 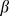 curve and cyan high 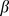 curve respectively). The high end of each curve corresponds to a limit beyond which the cell can not sustain its plasmid population due to a negative cell growth rate that leads to cell death. Points marked with circles on the medium 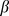 curve (green) represent equilibrium copy numbers; a filled circle indicates stability and the existence of a characteristic copy number, whereas an open circle indicates an unstable equilibrium. The critical 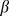 curve (red), which is tangential to the diagonal, represents the limit (edge) of stability, in which the stable and unstable characteristic copy numbers collapse to a singular point.

We begin by focusing our attention to a single cell that contains a population of plasmids that are identical with respect to their replication profile. We initially ignore the stochastic nature of replication and cell division and consider the copy number to be a continuous variable, with both the host growth and the copy number described by deterministic differential equations (see Equations 1 and 2 in Supplementary Text S1). Our model allows us to establish a relationship between the number of plasmids at the beginning of the cell cycle (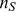) and the number of plasmids at the end of the cell cycle at the time of cell division (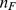), where 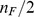 represents the number of plasmids in the resulting daughter cells, assuming equipartitioning during cell division. Figure 2 demonstrates a typical relationship between 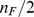 and 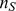, which is dependent on the plasmid replication rate which is, in turn, a function of the plasmid parameters 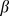, 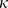 and 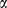 (see Equation 2). Subject to the initial number of plasmids in the parent cell 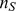, the resulting daughter cells will have either fewer (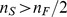), more (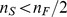) or as many plasmids (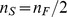) as their parent cell initially contained. The latter case represents the cross-generational equilibrium of a particular copy number for a given set of plasmid parameter values. This equilibrium can be either stable or unstable, depending upon the response of the system to fluctuations in copy number. The conditions for a stable equilibrium where there is a *stable characteristic copy number* 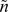 can be defined according to:
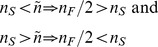
(3)so that when a cell has fewer than 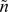 plasmids at the beginning of the cell cycle, plasmids over-replicate and when it has more than 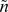 plasmids, plasmids under-replicate. In both cases, which can emerge as a result of stochasticities in plasmid replication or segregation upon cell division, the tendency is towards an equilibrium future cell cycle with 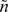 plasmids again. An example of a stable characteristic copy number is the point marked with a filled circle in Figure 2. The point marked with an open circle on the same curve is an unstable equilibrium copy number, any perturbation to which will lead either to plasmid over-replication or under-replication, the latter case in this instance resulting in movement towards the stable equilibrium.

### The Boundaries of Plasmid Stability

**Figure 3:**
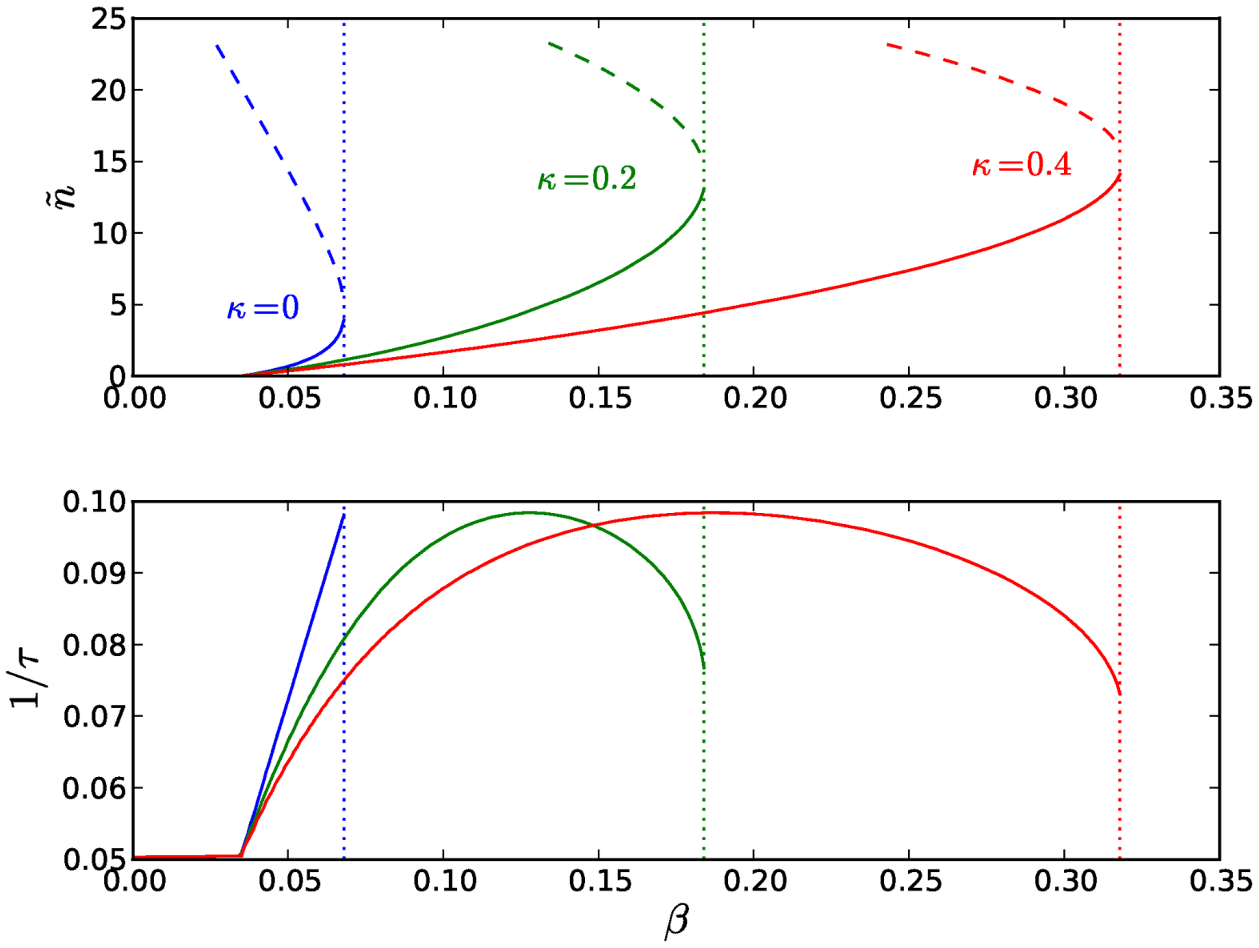
Obedience (affinity to inhibitor) ensures copy number stability. Stable (solid) and unstable (dashed) equilibrium copy numbers 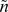 for a single cell as a function of basal plasmid replication rates 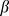 for 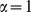 and various values of the binding affinity 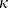 (blue for 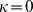; green for 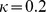; red for 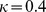). The values of cell fitness (calculated as the reciprocal of the cell division time 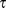) that correspond to the stable characteristic copy numbers are shown in the bottom panel. The stable and unstable equilibrium copy numbers collapse to a singular point (the edge of copy number stability as demonstrated by the critical 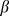 curve in Figure 2) that is marked by a colored vertical dotted line, beyond which there exist no characteristic copy numbers, i.e. plasmids over-replicate for any initial copy number. The limit below which the stable characteristic copy number 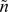 becomes zero is 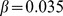. In the case where 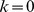 (blue lines), the edge of stability coincides with maximum cell fitness; for 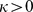 (green, red lines), the host fitness peak is surrounded by suboptimal parameter configurations which are characterized by stability with respect to copy number.

For any configuration of plasmid replication parameters 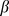, 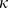 and 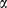, we can determine whether a stable characteristic copy number exists, what its value is, and what is the corresponding cell fitness, which is defined as the reciprocal of the time 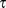 required for a cell to divide. Figure 3 presents the results of exploring the space of plasmid parameters for two distinct CNC regimes. First, we consider the case in which plasmids have self-determined replication rates independent of the presence of other plasmids in the same host (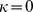, NO-CNC). In this case, the only mechanism of controlling the copy number is plasmid self-restraint (in the form of 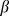), what early theoretical approaches termed “passive” copy number control [36]. Second, we consider the case of an active negative feedback loop between copy number and plasmid replication rate that is realized through the synthesis of trans-acting replication inhibitors (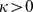, with 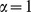, CNC).

In the former case (NO-CNC), we observe that the system has a stable non-zero characteristic copy number for an extremely limited region of basal plasmid replication rates 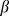. This stable region is surrounded by regions of plasmid instability characterized by consistent under- or over- replication of plasmids for any initial copy number (described by the low and high 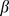 curves in Figure 2). Within the region of plasmid stability, cell fitness increases with 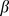 until a critical point which marks the transition to instability where plasmids consistently over-replicate. Hence, under no CNC, inter-cellular selection would favor increasing values of the plasmid replication rate (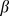), since this translates to higher cell fitness, until the critical point of plasmid instability is reached. This transition (denoted by a vertical dotted line in Figure 3) occurs at maximum cell fitness and is characterized by the collapse of the stable and unstable equilibrium copy numbers to a singular copy number 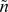 (an event that is captured by the critical 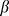 curve in Figure 2). Consistent over-replication results in future cell cycles with an increasing number of plasmids which leads, eventually, to plasmid explosion and cell death. The activation of the CNC system (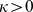, with 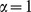) effectively widens the range of plasmid stability and, crucially, decouples the point of transition to instability from the point of optimal cell growth. As a result, the configuration of plasmid replication parameters that yields optimal cell growth is now surrounded by suboptimal, yet also stable, regions. Under these conditions, inter-cellular selection for increased cell division rates would favor cells containing plasmids with stable copy numbers.

### Plasmid Stability and Host Growth

Our previous simulations demonstrated that, when stochasticity is ignored, there is only a limited range of 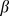 that allows for stability in plasmid replication. In the absence of CNC, optimization of the cell division rate would result in plasmid replication rates at the edge of stability, whereas the presence of CNC both broadens the range of stable replication rates, as well as creates a situation in which optimal cellular fitness is located in the interior of this region of stability. We now proceed by extending the deterministic unicellular framework we have considered so far in order to investigate plasmid stability and host performance in the context of stochastic multicellular simulations that describe the asynchronous growth and division (or death) of hosts and the autonomous replication of plasmids within such hosts. We introduce stochasticity in plasmid replication by considering the expected number of replication events for plasmids in each host at each discrete time point to be Poisson-distributed, as specified by Equation 2 (for more details see Supplementary Text S1).

**Figure 4:**
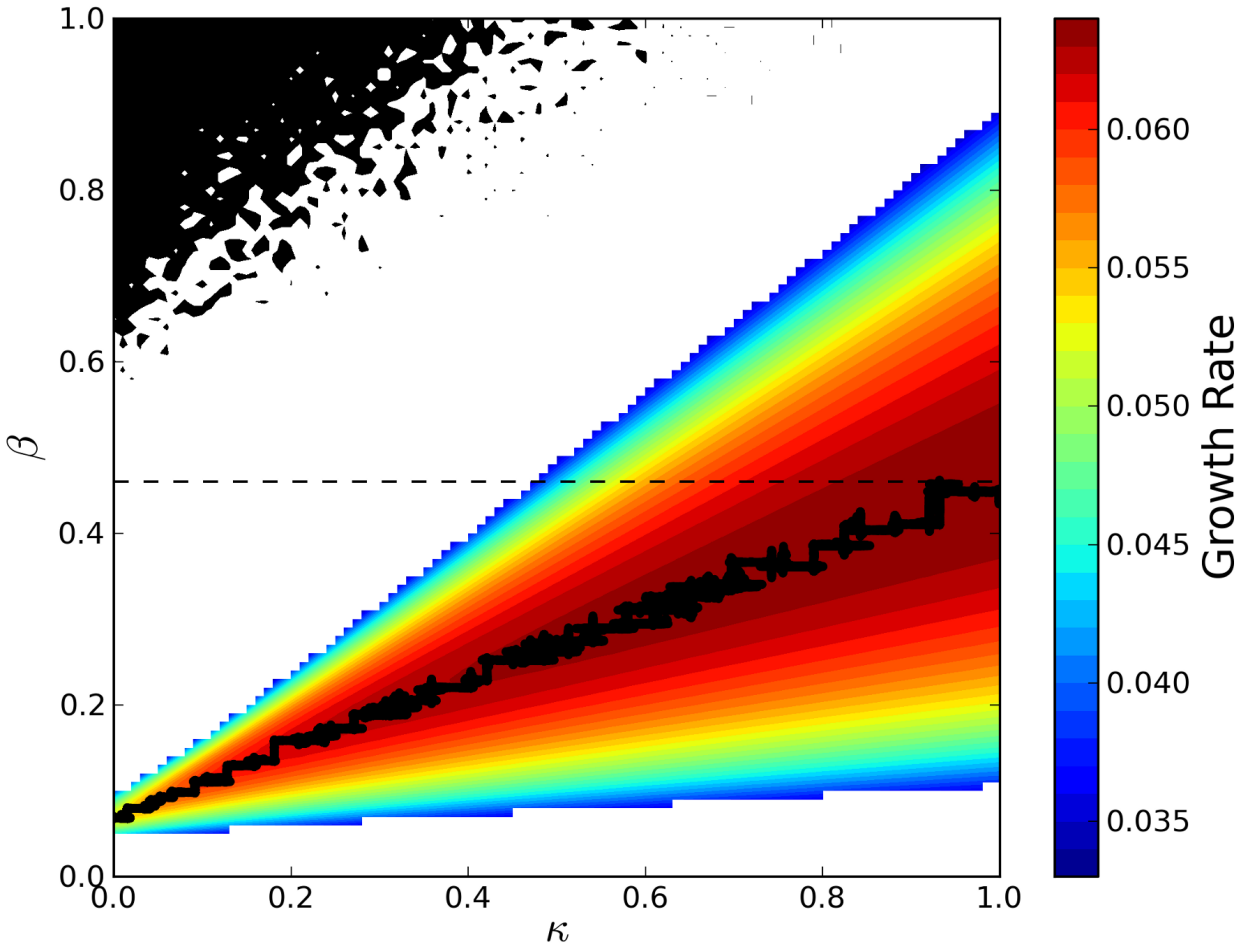
Co-evolution of obedience and baseline replication, in face of constant policing. The heatmap displays the population's net average growth rate (expressed as the difference between the average division and death rates) as a function of the plasmid replication parameters 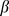 and 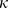, averaged across three independent stochastic multicellular simulations with plasmid homogeneity (no mutations) and a fixed rate 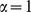 of inhibitor production. The white areas below and above the stable region represent the regions of the plasmid parameter space in which plasmids are eliminated from the population due to consistent under- or over- replication respectively. The black clusters in the upper-left corner represent the collapse of the host population under the weight of excessive plasmid replication. The horizontal dashed line indicates the value of 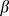 for which the system reaches optimal growth at 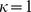 (see Figure 5). Finally, the black ladder-like path outlines the evolution of CNC in a stochastic simulation where 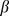 and 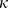 are allowed to mutate with probability 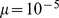 and a fixed rate 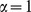 of inhibitor production.

The performance of a particular strain in such a stochastic simulation, in which hosts are infected by plasmids with identical replication parameter values, can be evaluated by calculating the average net host growth rate as the difference between the average host division and death rates. Figure 4 displays the resulting fitness landscape of independent strains as a function of the corresponding values of the plasmid replication parameters 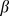 and 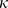 given 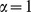. We note that, due to the homogeneity of the plasmid population, parameters 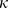 and 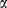 are interchangeable (see also Equation 2) and, as such, the fitness landscape of 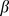 and 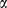 given 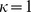 is identical to the fitness landscape of 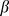 and 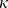 given 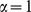. The region of plasmid stability in this landscape is dominated by the gradient of the net host growth rate leading to an area of optimal growth in which obedience to policing is maximally strong (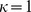). Just as in the unicellular deterministic case, the stable region is surrounded by regions of plasmid instability, in which plasmids are eliminated from the host population, due to the absence of a stable characteristic copy number 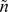 and the consistent under- or over- replication of plasmids. Consistent under-replication leads to the gradual dilution and eventual disappearance of plasmids from the population (white area below the stable region in Figure 4). Consistent over-replication leads to an elevated copy number that slows down cellular growth (see also Equation 1), providing more time for plasmids to replicate, thereby further compromising cellular growth. As such, plasmid-free hosts, resulting from stochastic segregational errors, are able to outgrow over-infected hosts, until the population is completely plasmid-free (white area above the stable region in Figure 4). In the case of extreme plasmid selfishness (at high 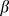, low 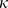), the host population collapses under the weight of excessive plasmid over-replication, before plasmid-free segregants are given the chance to outgrow over-infected hosts and form a plasmid-free population (black clusters in Figure 4).

### Levels of Obedience and the Efficiency of Replication Control

**Figure 5:**
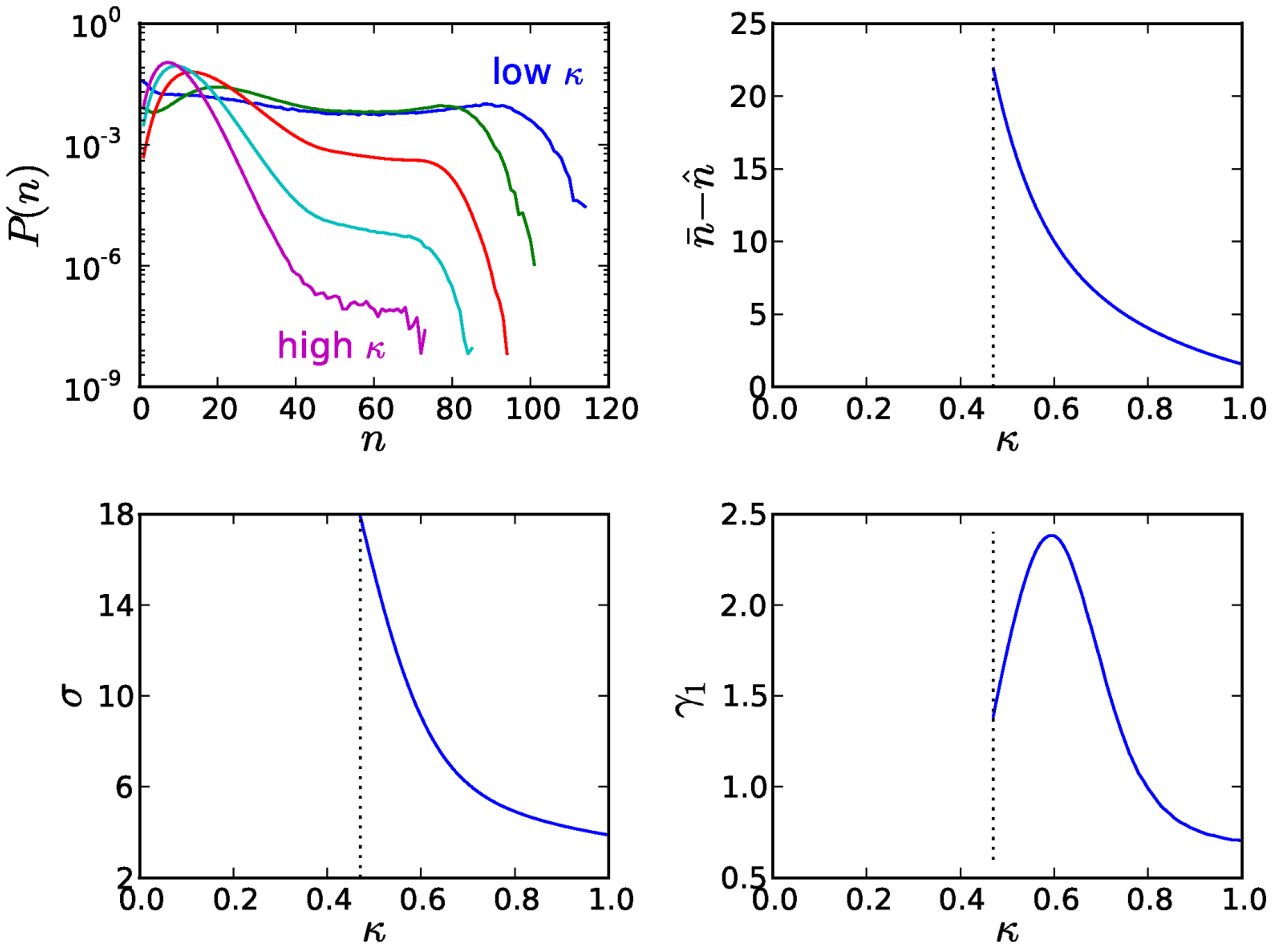
The influence of obedience on plasmid copy number distributions. The effects of obedience (increasing copy number control 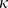) on the distribution of plasmid copy numbers for a fixed value of the plasmids' basal replication rate 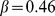, chosen so that the population fitness for this value is optimal at maximal CNC (see horizontal dashed line in Figure 4). The weak CNC regime (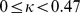) is unstable and plasmids are eventually eliminated from the population. Copy number distributions are given for different values of 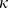 (top left; blue for 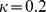, green for 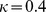, red for 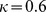, cyan for 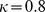 and magenta for 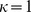). The distributions have been calculated by recording the copy numbers of all plasmid-infected hosts over the entire course of a multicellular stochastic simulation. The discrepancy between the average copy number 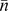 and the copy number 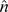 that is optimal for host growth is given as a function of 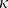 (top right), as well as the standard deviation 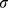 (bottom left) and skewness 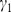 (bottom right) of the copy number distributions.

The stochasticities in plasmid replication and segregation upon cell division give rise to a distribution of copy numbers in the population that occur across all plasmid-infected hosts over the course of a simulation. We explored the effects of obedience (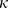 or policing 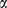, since these are interchangeable due to the homogeneity of the plasmid population) on the features of these distributions, by considering a cross-section of the fitness landscape for a fixed value of the plasmids' basal replication rate 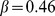, which corresponds to the optimal net growth rate at maximal CNC (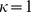). The weak CNC regime along this cross-section of the fitness landscape (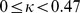) is unstable and plasmids are eventually eliminated from the population (see Figure 5). The broadness of the copy number distributions in this regime reflects the extensive variation and drift of copy numbers in the population, due to the amplification of stochastic copy number fluctuations [15]. The transformation of the distributions begins at the intermediate range of obedience (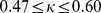), with the emergence of a clear peak; nevertheless, the presence of a heavy tail indicates the persistence of plasmid replication instabilities. These instabilities are minimized in the region of strong CNC (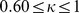) as the distributions become progressively less skewed, due to the increasing efficiency in controlling stochastic copy number fluctuations and the corresponding reduction in copy number variation. At the same time, the discrepancy between the distributions' average copy number 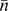 and the copy number 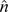 that is optimal for host growth (see also Equation 1) becomes lower with increasing obedience 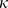, thus inducing an acceleration of the net average host growth up to the optimal rate at maximal CNC (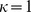).

### The Evolution of Collective Restraint

Having explored the effects of homogeneous plasmid cooperation on host growth and plasmid stability, we now ask how these effects influence the evolution of the plasmid replication parameters in the broader context of the conflict between the levels of selection. To this end, we introduce plasmid variation in our multicellular stochastic simulations: each plasmid replication event implies the possibility of mutation with probability 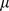, in which case the value of exactly one of the plasmid's replication parameters, chosen at random with equal probabilities, is modified.

Starting with an initial population of plasmids that do not respond to inhibitors (i.e. 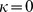), we allow 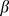 and 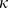 to evolve given a fixed rate of inhibitor production 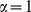. The resulting evolutionary dynamics, shown in Figure 4, demonstrate the emergence of efficient replication control as driven by the synergies between intra-cellular selection favoring immediate plasmid reproductive gains (higher selfishness 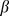, lower obedience 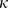), and inter-cellular selection favoring evolutionary adjustments towards those regions of the plasmid parameter space where the net host growth rate increases. In effect, and due to the transient and epigenetic nature of stochastic copy number fluctuations, inter-cellular selection operates upon the net host growth rate accumulated over a few generations and, therefore, upon the copy number distributions associated with particular configurations of the plasmid replication parameters [27]. As such, the evolutionary adjustments favored by inter-cellular selection come in the form of cooperative plasmid parameter mutations, such as increased plasmid self-restraint (lower selfishness 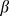) or increased sensitivity to the inhibitor (higher obedience 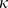), which alter the mode of plasmid replication so as to ensure a reduction, first, in the discrepancy between the optimal 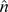 and mean 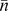 copy numbers, and, second, in the magnitude of copy number fluctuations (i.e. the variation of the copy number distribution). This way, the evolution of CNC unfolds with an escalating succession of selfish and cooperative plasmid parameter mutations that develops along the gradient of the host fitness landscape, leading the system towards the region of optimal host growth at maximal CNC. Further escalation is prevented due to the limits that are imposed on plasmid parameter values; the absence of such limits would yield a ratcheting effect whereby the succession of selfish and cooperative mutations would continue indefinitely, limited only by the costs of producing the corresponding factors involved in plasmid replication (initiators and inhibitors).

**Figure 6:**
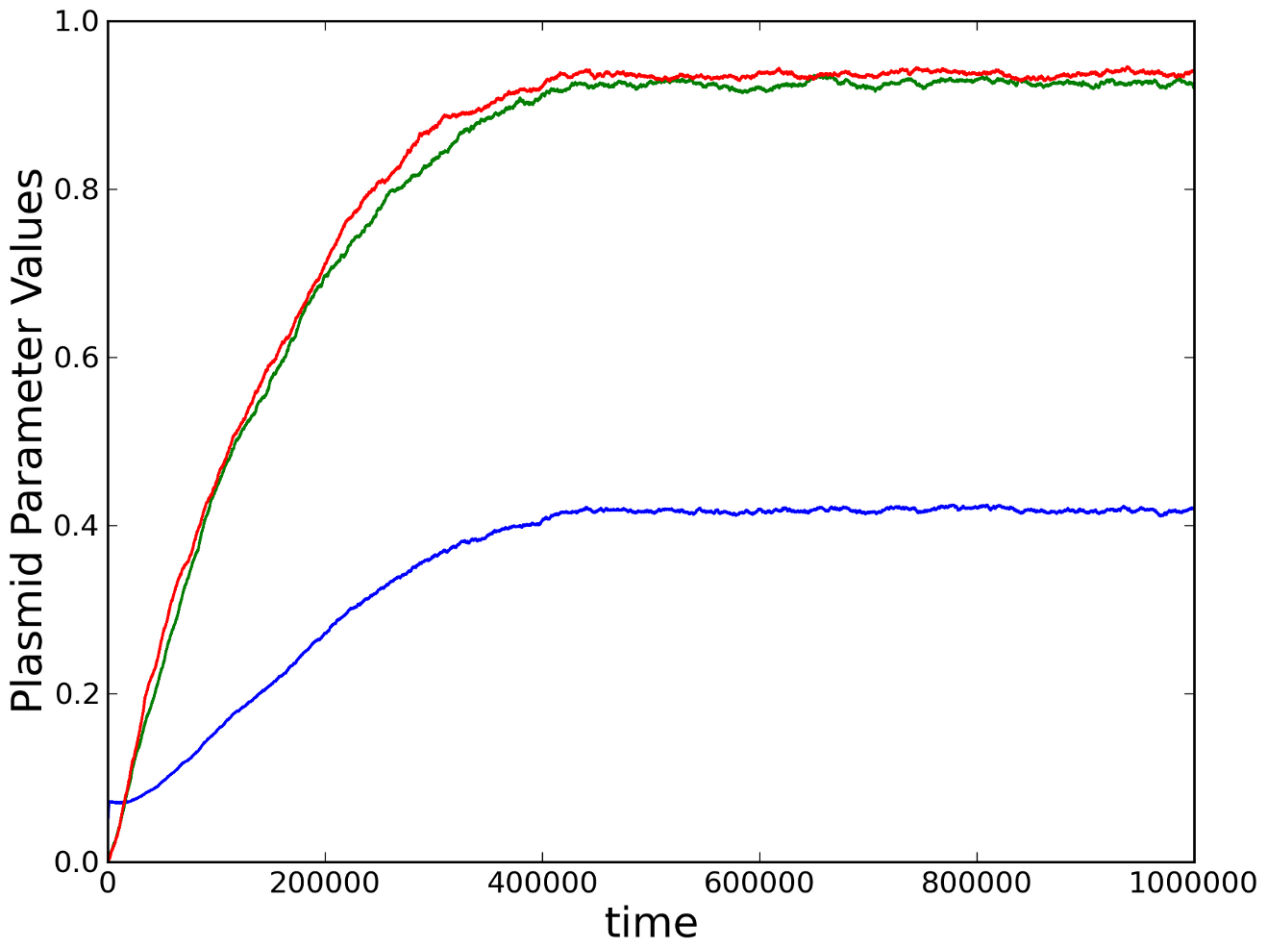
The co-evolution of policing, obedience and baseline reproduction. Average plasmid parameter values 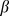 (blue), 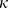 (green) and 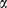 (red) over time, showing the evolution of the CNC mechanism in stochastic multicellular simulations, with mutation probability 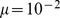. Results have been averaged across 50 independent simulations with the same initial conditions.

The same cooperative outcome (evolution of an efficient CNC system) is obtained when we allow all three plasmid replication parameters 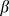, 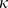 and 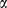 to evolve from an initial state where plasmids neither produce (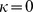) nor respond (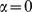) to inhibitors (see Figure 6). In this case, cooperative mutations can be either cis-specific (higher obedience 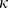) as before, or trans-specific (greater policing 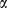), in which case a mutation that increases the rate 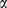 of inhibitor production by an individual plasmid will influence not just the mutant but all plasmids in the intra-cellular replication pool (due to the 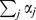 term in Equation 2). The cis-specificity of 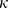 implies that a cooperative mutation inducing a higher sensitivity to the inhibitor is costly at the intra-cellular level, since it decreases the mutant's chances of immediate reproductive success in the replication pool. The trans-specificity of 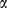 introduces a coercive element to cooperation, because the production of inhibitors regulates the replication of all plasmids in the pool. It also creates the potential for subversive plasmid strategies according to which individual plasmids can gain an advantage in the replication pool by zealously producing the inhibitor (high policing 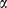) while maintaining a low sensitivity (obedience 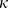) to that inhibitor themselves. Nevertheless, this scope for opportunistic behavior does not prevent the emergence of the policing CNC mechanism. In fact, we find that plasmid parameter variation within cells is quite low (see Table 1), so that hosts are inhabited by a, more or less, homogeneous plasmid population (i.e. plasmids are highly related to their intra-cellular neighbors), due to the lack of plasmid migration (horizontal transmission) between hosts. The degree of plasmid homogeneity is reduced by approximately an order of magnitude between hosts, compared to its value within hosts, thus generating the host growth differential upon which inter-cellular selection operates by favoring stricter control over plasmid replication.

**Table 1:** Average plasmid parameter variation.

	within hosts	between hosts
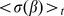	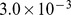	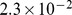
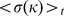	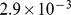	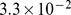
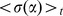	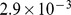	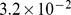

The table summarizes the intra-cellular (within hosts) and inter-cellular (between hosts) variation of plasmid replication parameters, represented as the standard deviation 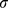, averaged over time 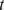 and across 50 independent stochastic multicellular simulations (the corresponding plasmid parameter averages are shown in Figure 6). The intra-cellular variation is calculated for all plasmids within a host with respect to the host's intra-cellular mean and is averaged across all hosts in the population. The inter-cellular variation is calculated for the intra-cellular means of all hosts with respect to the population's (global) mean.

### The Effects of Policing Costs on the Evolution of CNC

**Figure 7:**
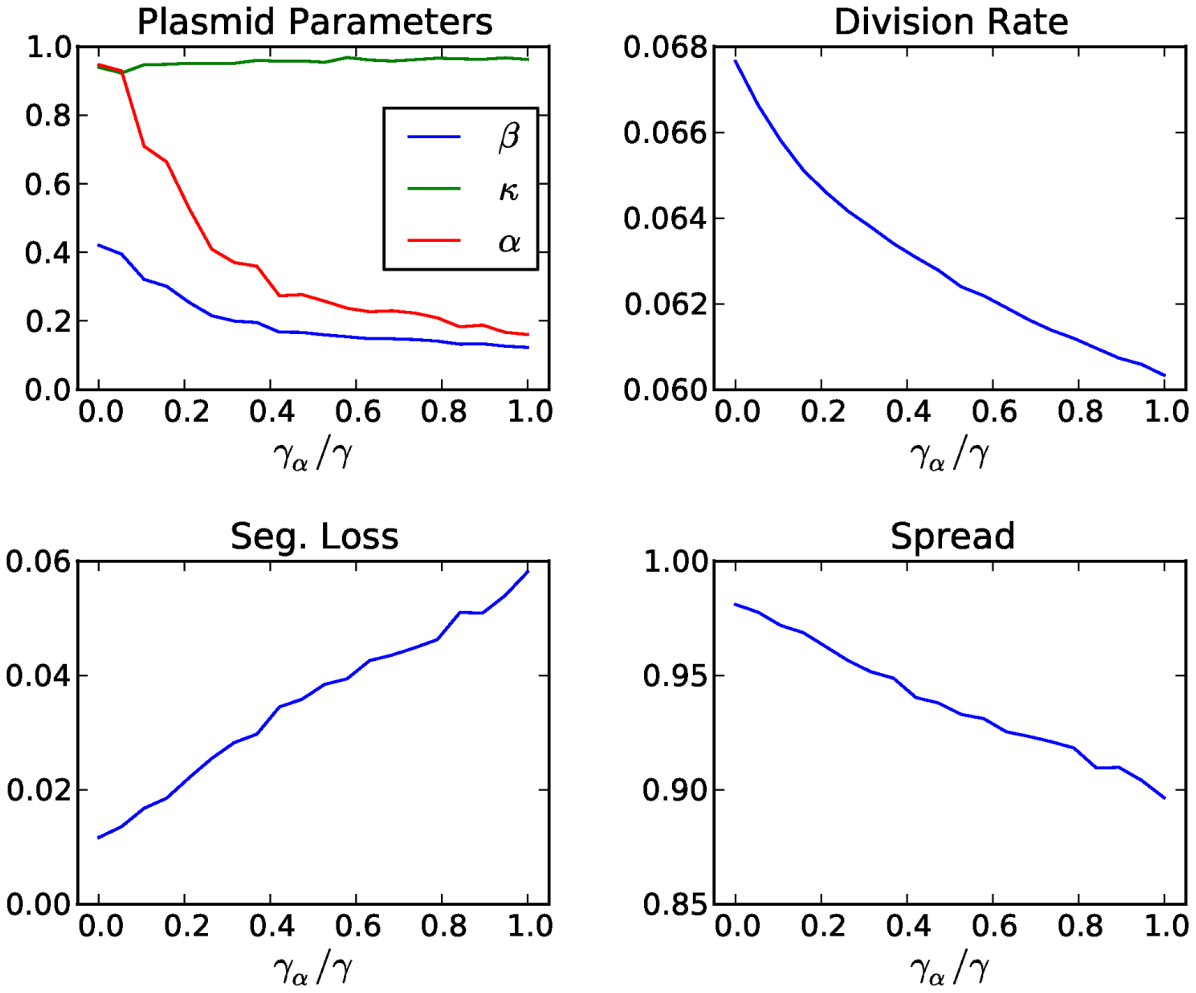
The effects of policing costs on performance metrics and the evolution of CNC. Average values of the plasmid parameters (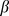, 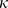, 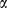), the rates of host division and segregational loss, as well as the fraction of plasmid-infected hosts in the population (plasmid spread) for varying costs of policing. The latter is expressed here as the production cost 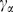 per unit of inhibitor relative to the constant general cost of maintenance 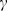 per plasmid copy (e.g. the cost of gene expression, replication etc.).

We also investigated the influence of policing costs to the evolution of collective restraint and the overall performance of the population, by introducing an additional cost term 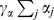 in Equation 1, where 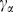 is the cost of production per unit of inhibitor paid by the host. This implies that there is now selection at the inter-cellular level against the production of policing resources, due to the associated policing costs that slow down host growth. Figure 7 shows that the increase in policing costs corresponds to a decrease in the production of policing resources (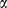), but not a collapse of plasmid obedience (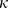) to policing. On the contrary, obedience is not only sustained but also slightly increases with rising policing costs. At the same time, the basal plasmid replication rate (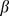) decreases so as to compensate for the gradual reduction in the availability of policing resources (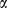). As a result, the CNC system remains functional throughout (since there is still selection at the inter-cellular level for high obedience 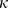) but becomes less efficient with increasing policing costs and the performance of the population deteriorates with lower division rates for hosts and higher rates of segregational loss for plasmids (see Figure 7).

### Comparisons between Individual and Collective Restraint

**Figure 8:**
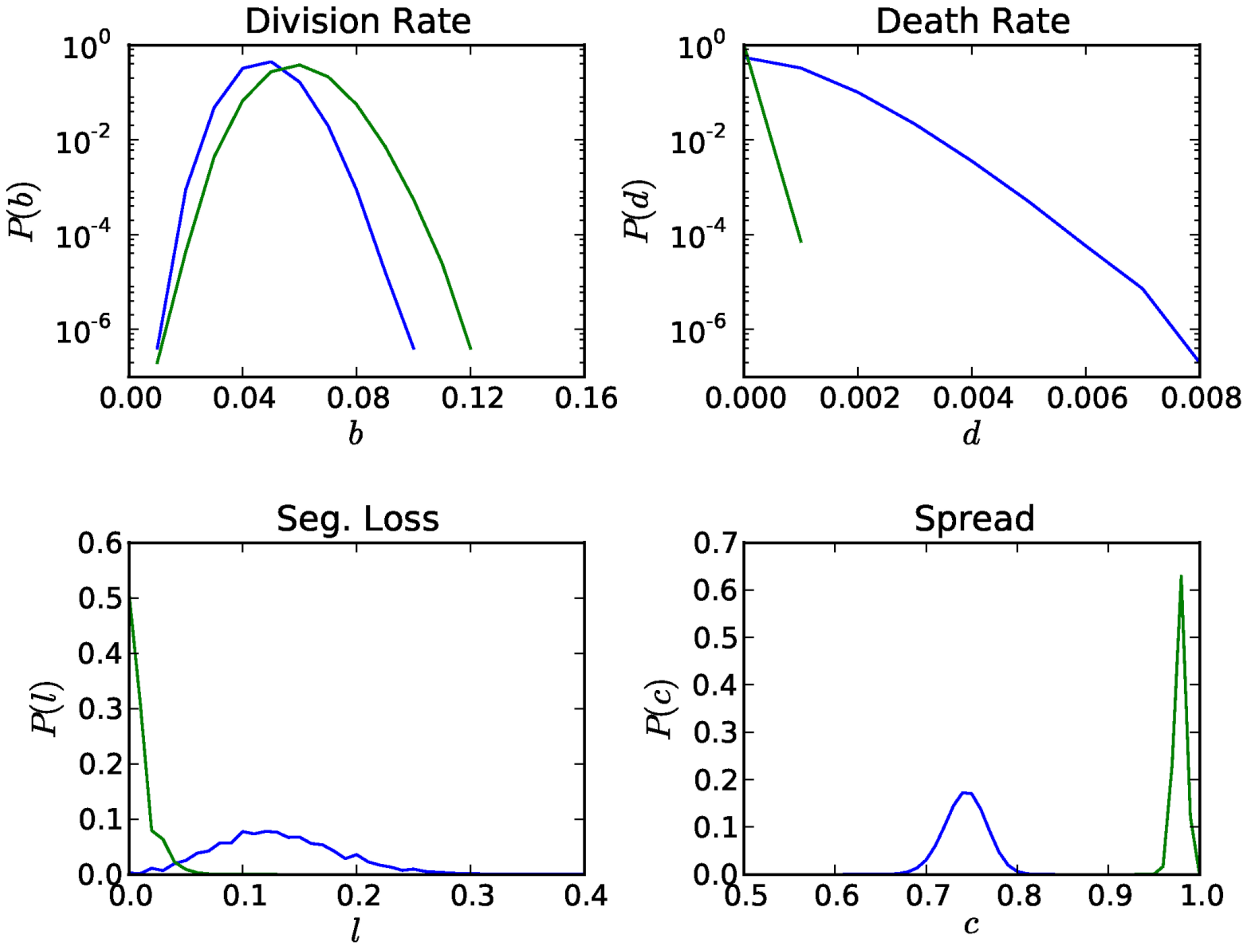
The effects of CNC on host and plasmid performance. Host performance is measured in terms of the division (top left) and death rates (top right), while the performance of plasmids is measured in terms of the segregational losses (bottom left) and the fraction of plasmid-infected hosts in the population (bottom right). The rate of segregational loss is calculated by recording at every time step the instances of a plasmid-free daughter cell arising from a plasmid-infected parent cell among all divisions of plasmid-infected cells. Comparisons are drawn between the baseline model in which policing is absent (NO-CNC in blue color; 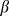 evolves, 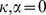) and the model in which policing and obedience are allowed to evolve (CNC in green color; 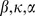 evolve). The distributions were calculated using the values of the last 100,000 steps of 50 independent stochastic multicellular simulations with the same initial conditions and mutation probability 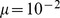.

The positive effects of CNC are not limited to hosts but extend to plasmids as well. We evaluated the advantages of CNC for hosts and plasmids by comparing the results of our multi-cellular stochastic CNC simulations (CNC; 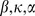 evolve) to those of the baseline model where policing is absent and plasmids replicate independently of the presence of other plasmids in the same host (NO-CNC; 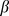 evolves, 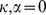). Host performance was evaluated in terms of the average division and death rates, while the performance of plasmids was assessed on the basis of the fidelity of vertical transmission and the spread of plasmids in the host population. Figure 8 demonstrates that all measures were significantly improved when CNC was functional (CNC simulations), compared to the case where the CNC mechanism was absent (NO-CNC simulations). As such, the beneficial effects of the CNC mechanism on plasmid stability, due to the stricter control of stochastic copy number fluctuations, allow for widespread host infection and the minimization of segregational losses within the margins allowed by inter-cellular selection, thus solidifying the persistence of the plasmid lineage in the population.

Finally, we also investigated the persistence and stability of an established policing mechanism among plasmids against invasion by selfish individuals, i.e. plasmids that are insensitive to replication inhibitors and replicate independently of the presence of other plasmids in the same host. More specifically, we simulated the competition between the NO-CNC (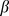 only with 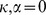) and the CNC (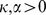) types (a) by mixing both types equally within hosts (within-host heterogeneity) and (b) by distributing the two types separately and equally among different hosts in the population (between-host heterogeneity). In every case that we examined, we observed the rapid displacement of the selfish type from the population. The complete prevalence of the CNC type demonstrates the robustness and stability of the mechanism of collective restraint in the face of invasion by selfish elements that bypass the policing mechanism in order to gain a relative advantage in the intra-cellular replication pool.

## Discussion

The dependence of vertically transmitted plasmids upon their hosts for survival and propagation mediates the reconciliation of two opposing forces, namely the plasmids' drive towards recklessness with respect to replication, which is favored by intra-cellular selection, and the host's requirement for an optimal configuration of metabolic benefits and burdens, which is favored by inter-cellular selection. The coupling between the levels of selection in our model is defined in terms of the influence of plasmid copy number on host growth; the latter depends on the mode of plasmid replication which, in turn, is a function of the intra-cellular plasmid replication parameters 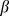, 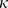 and 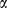 (see Equation 2). The region of stability in the plasmid parameter space is defined by the existence of stable characteristic copy numbers 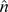 (see Equation 3). In the absence of CNC (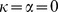), where plasmids replicate independently of the presence of other plasmids in the same host, the edge of plasmid stability coincides with optimal host growth. In an evolutionary context, this implies that plasmids will evolve to the edge of plasmid stability driven by intra-cellular selection (which favors higher 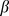) and this drive will be consistent with selective forces at the inter-cellular level since host fitness is also increasing. The transition to instability stimulates the conflict between the levels of selection as net host growth slows down due to the increasing metabolic costs resulting from the consistent over-replication (lack of inter-generational stability) of the selfish mutants. The activation of the CNC system (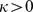, 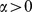) mitigates the detrimental effects of these transient tensions between the levels of selection by expanding the region of plasmid stability so that the plasmid parameter configuration that yields optimal growth no longer coincides with the edge of plasmid stability.

CNC is realized by cooperating plasmids that participate in the policing mechanism that they themselves construct and maintain. This introduces a collectivist element to the repression of competition in the intra-cellular replication pool, due to the trans-specificity of the plasmid-coded replication inhibitors. Increasing cooperation through collective restraint has beneficial effects on hosts and plasmids alike, by strengthening the system's defenses against copy number fluctuations resulting from stochasticities in plasmid replication and segregation upon cell division. These fluctuations are reflected in the copy number distributions, whose variation decreases with increasing CNC (see Figure 5). As a result, hosts grow faster and plasmids maximize their spread in the population and minimize their loss due to segregational errors, within the margins allowed by inter-cellular selection. The landscape of population growth, expressed as a function of the plasmid replication parameters, is dominated by a gradient that leads progressively to the region of optimal growth at maximal CNC (see Figure 4). The synergies between the levels of selection are reflected in the co-evolution of the plasmid replication parameters that develops along this gradient and unfolds with a succession of selfish and cooperative plasmid mutations that drive the system to its optimal state at full cooperation. Along this gradient, we find a most accurate alignment between the average copy number 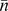 (a proxy for the characteristic copy number 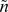) and the copy number 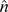 that is optimal for host growth. We expect the accuracy of this alignment to decline when plasmids adopt migratory strategies such as conjugative transfer, the horizontal nature of which undermines the strong mutualistic nature of the host-symbiont relationship.

The regulation of plasmid replication by means of the CNC mechanism is characterized by a more general trade-off between the immediate reproductive gains of an individual and the longer-term success of the collective to which that individual belongs; the latter can only be improved if the urge to satisfy the former can somehow be repressed. To this end, the repression of competition in the plasmid replication pool is achieved through the obedience (binding affinity) of plasmids to the policing resource (replication inhibitor) that they themselves produce. Hosts in which plasmid obedience to policing is strong or under development, will outgrow fellow hosts in which obedience is weaker or absent, thus motivating the reinforcement of cooperation and the eventual establishment of the CNC mechanism.

## Supporting Information

Text S1 Detailed information about the plasmid profile used as well as about the methods and their implementation (i.e. the unicellular deterministic and multicellular stochastic simulations).
(PDF)
