# Supplementary material for: The Evolution of Collective Restraint: Policing and Obedience among Non-conjugative Plasmids
Source: PLoS Comput Biol. 2013 Apr 18;9(4):e1003036. doi: 10.1371/journal.pcbi.1003036 (PMC3630227; doi:10.1371/journal.pcbi.1003036)
Supplement: Text S1 — Detailed information about the plasmid profile used as well as about the methods and their implementation (i.e. the unicellular deterministic and multicellular stochastic simulations). (PDF) [file pcbi.1003036.s001.pdf]

Supplementary Text S1

1 Methods

1.1 Plasmid Profile

We consider a single baseline plasmid type throughout our simulations, characterized by its contribution to host biomass growth as a function of the copy number according to Equation 1 (in main paper). This contribution is specified by the plasmid parameters $\phi = 0.1$, $\lambda = 3$ and $\gamma = 3 \times 10^{-3}$ and gives rise to a copy number that is optimal for host growth $\hat{n} = 7$. The basal host growth rate is $\omega_0 = 0.05$.

1.2 Unicellular Deterministic Simulations

We consider a single cell and examine its individual growth, as well as the growth of the contained plasmid population. If we assume the plasmid population in the cell to consist of identical plasmids with respect to their replication parameters β, κ and α, and the copy number n to be a continuous variable, then we can consider the following deterministic system of coupled differential equations:

$$\frac{d\Omega}{dt} = \omega_0 + \frac{\phi n}{\lambda + n} - \gamma n \tag{1}$$

$$\frac{dn}{dt} = \frac{\beta n}{1 + \frac{\kappa \alpha n}{\Omega}} \tag{2}$$

The numerical integration of this system until cell division ($\Omega \geq 2\Omega_0$) or death ($\Omega \leq 0$), with the initial conditions $\Omega_0 = 1$ and $n = n_S$, will yield the number n_F of plasmids at the end of the cell cycle, as well as the time τ it took for the cell to divide. The reciprocal of the division time $1/\tau$, given that the cell has indeed divided, constitutes a measure of cell fitness.

1.3 Multicellular Stochastic Simulations

We consider a population of hosts which grow and divide or die and impose a fixed maximum population capacity ($N = 1000$), so that, when the population operates below that capacity, daughter cells are added to the growing population. When the population operates at maximum capacity, the two daughter

cells resulting from a potential cell division replace the parent cell and a randomly selected cell from the population so that, at maximum capacity, every birth (cell division) corresponds to a random death. We assume that plasmids do not encode an explicit partitioning (`par`) mechanism, so that when a cell divides, its plasmids are distributed among daughter cells on the basis of binomial segregation (i.e. each plasmid is equally likely to to end up in either of the daughter cells). Each cell's basal growth rate ω_0 (see Equation 1 in paper) is defined as a random variable that is meant to capture the stochastically fluctuating environmental conditions, whose effects can either promote or impede host growth. The variable follows a Gaussian distribution with mean 0.05 and standard deviation 0.01 and, once initialized upon cell birth, it remains unchanged throughout a cell's life cycle.

The replication of a plasmid implies the possibility of mutation with probability μ, in which case exactly one of its parameter values (β, κ or α), chosen at random, is modified. Let $v \in [v_0, v_1]$ be the value of the parameter to be mutated, where $v_0 = 0$ and $v_1 = 1$ are the minimum and maximum allowed values of the parameter. We choose the new parameter value v' from a uniform distribution in $\big[\max\{v_0, v - v_d\}, \min\{v_1, v + v_d\}\big]$, where $v_d = 0.05$ determines the maximum possible deviation in value that mutations are allowed to make.
